# Genome-wide analysis of the mulberry (*Morus abla* L.) GH9 gene family and the functional characterization of MaGH9B6 during the development of the abscission zone

**DOI:** 10.3389/fpls.2024.1352635

**Published:** 2024-04-03

**Authors:** Jing Deng, Bilal Ahmad, Xuan Deng, Zelin Fan, Lianlian Liu, Xiuping Lu, Yu Pan, Xingfu Zha

**Affiliations:** ^1^ State Key Laboratory of Resource Insects, Southwest University, Chongqing, China; ^2^ National Key Laboratory of Tropical Crop Breeding, Shenzhen Branch, Guangdong Laboratory of Lingnan Modern Agriculture, Key Laboratory of Synthetic Biology, Ministry of Agriculture and Rural Affairs, Agricultural Genomics Institute at Shenzhen, Chinese Academy of Agricultural Sciences, Shenzhen, China; ^3^ College of Horticulture and Landscape Architecture, Southwest University, Chongqing, China

**Keywords:** GH9 gene family, abscisic acid, 2, 4-D, *Morus alba* L, abscission

## Abstract

Plant glycoside hydrolase family 9 genes (GH9s) are widely distributed in plants and involved in a variety of cellular and physiological processes. In the current study, nine GH9 genes were identified in the mulberry and were divided into two subfamilies based on the phylogenetic analysis. Conserved motifs and gene structure analysis suggested that the evolution of the two subfamilies is relatively conserved and the glycoside hydrolase domain almost occupy the entire coding region of the GH9s gene. Only segmental duplication has played a role in the expansion of gene family. Collinearity analysis showed that mulberry GH9s had the closest relationship with poplar GH9s. *MaGH9B1, MaGH9B6, MaGH9B5*, and *MaGH9B3* were detected to have transcript accumulation in the stalk of easy-to drop mature fruit drop, suggesting that these could play a role in mulberry fruit drop. Multiple cis-acting elements related to plant hormones and abiotic stress responses were found in the mulberry GH9 promoter regions and showed different activities under exogenous abscisic acid (ABA) and 2,4- dichlorophenoxyacetic acid (2,4-D) stresses. We found that the lignin content in the fruit stalk decreased with the formation of the abscission zone (AZ), which could indirectly reflect the formation process of the AZ. These results provide a theoretical basis for further research on the role of GH9s in mulberry abscission.

## 1 Introduction

Glycoside hydrolase family 9 (GH9) is prevalent in plants where it comprises enzymes capable of catalyzing the hydrolysis of glycosidic bonds. Some GH9 members (also knowns as cellulase) are linked to various aspects of plant growth, cell wall metabolism, and cellulose biosynthesis (Rose and Bennett, 1999; Bhandari et al., 2006; Shani et al., 2006). Cellulolytic activity is linked to cell wall construction during cell expansion, cell wall disassembly during fruit ripening and shedding (del Campillo, 1999; Rose and Bennett, 1999; Molhoj et al., 2002), and cellulose degradation (Nicol et al., 1998). Members of the GH9 family have been identified in many species, such as poplar (Du et al., 2015), *Arabidopsis* (Urbanowicz et al., 2007), and wheat (Luo et al., 2022). The plant GH9 family is generally divided into three subclasses (A, B, and C) (Henrissat, 1991; Davison and Blaxter, 2005). GH9A consists of a membrane-anchored protein (Ordin and Hall, 1968; Robert et al., 2005). GH9B consists of a secreted protein with only one catalytic domain (CD). Subfamily B usually contains signal peptides, most of which are secreted proteins and are the most abundant in the plant GH9 gene family with complex and variable functions. Lastly, GH9C has a CD and a C-terminal extended fibrin-binding domain (CBD). To date, the biological relationship of most GH9 members and the relationships between gene function, structure, and evolution are unclear. Silencing GH9B1 in *Arabidopsis thaliana* resulted in wrinkled cell walls with reduced cellulose and lignin content (Tsabary et al., 2003). In rice, co-expression of *OsGH9B8*, *OsGH9B9*, *OsGH9B10*, and *OsGH9B11* with cellulose synthase catalytic subunit genes *OsCESA4*, *OsCESA7*, and *OsCESA9* suggests that they play roles in secondary wall formation (Xie et al., 2013).

The abscission of plant organs is often accompanied by the division and degradation of cell walls. Higher synthesis and activity of cell wall hydrolases are observed in most abscission events. Two major cell wall hydrolases [cellulase and polygalacturonase (PG)] have been well studied in various plants for their involvement in the organ abscission process (Brummell et al., 1999; Gonzalez-Carranza et al., 2002). Many members of the GH9 gene family have key roles in flower and fruit drop. Certain GH9 genes were strongly expressed in *Arabidopsis* after the organ abscission process and played important roles in cell division during the latest stages of abscission (Lashbrook and Cai, 2008; Patharkar and Walker, 2018). The expression of two cellulase genes (*LcCEL2* and *LcCEL8*) was closely related to litchi abscission. Transgenic *Arabidopsis* overexpressing *LcCEL2* or *LcCEL8* showed marked premature abscission of floral organs compared with wild-type *Arabidopsis* (Li et al., 2019a). Transcripts of cellulase genes *CEL1* and *CEL2* accumulate in the avocado fruit abscission zone (AZ) and are involved in the abscission process (Tonutti et al., 1995).

Trees have unique biological characteristics that distinguish them from herbaceous species, including their large size, long generation intervals, and wood formation, which make it difficult to study the cellular and molecular mechanisms behind their growth and development (Tuskan et al., 2006). Mulberry (*Morus* spp.) is an economically important perennial woody plant that is rich in compounds such as anthocyanins (Veberic et al., 2015), alkaloids (Kim et al., 2013), and flavonoids, which play an important role in food and medicine. In mulberry, heavy fruit drop at the maturity stage is a serious problem that leads to reduced yield. Mulberry fruit drop can occur due to various reasons, but the formation of the AZ is considered the most significant one. Unfortunately, there are only a few studies exploring the basis of fruit drop in mulberry. Excessive fruit drop at the fruit maturity stage is among the leading problems of mulberry production. Identification of specific genes and breeding new crop varieties are among of the most effective means to improve the environmental adaptability of plants. A lot of research work has been done to cope with protecting mulberry from biotic and abiotic stresses. However, there is less information available about the molecular basis of a higher fruit drop at fruit maturity. According to the available literature, fruit abscission is one of the most important reasons of fruit drop. The process of plant organ abscission is generally divided into three stages. The first stage is the signal response stage, which includes developmental and environmental signals that can lead to the differentiation of AZ cells into the AZ structure. In the second stage, known as the regulation stage, plants become sensitive to ethylene and abscisic acid (ABA), but insensitive to auxin. At this stage, shedding-specific selection factors such as *IDA, DAB, F-box*, and protein kinase HAESA are activated. In the third and final stage of execution, there is an increased expression of cell wall loosening agents such as cellulases, polygalacturonases, β-galactosidases, and expansins (Sawicki et al., 2015; Gulfishan et al., 2019). GH9s have been shown to play important roles in the third stage of the abscission process (degradation of cellulose in plants and fungi) (Cantarel et al., 2009; Konar et al., 2022) and have been well studied in model plants like *Arabidopsis* and tomato. However, there is less information about the roles of GH9s in mulberry. To address this, we investigated the phylogenetic relationship, chromosome localization, gene structure, cis-elements, and conserved motifs of nine GH9 genes. Furthermore, we analyzed the subcellular localization of MaGH9B and how its gene behaves under ABA and 2,4-dichlorophenoxyacetic acid (2,4-D) stress. These results provide a foundation for further understanding the function of mulberry GH9 genes.

## 2 Materials and methods

### 2.1 Plant materials and stress treatments

The mulberry (*Morus alba* L.) and *Nicotiana benthamiana* seeds were taken from the State Key Laboratory of Resource Insects, Southwest University, Chongqing, China. In a phytotron, mulberry and tobacco seedlings were grown under specific conditions. They received 16 h of light and 8 h of darkness per day, the temperature was maintained at 24 ± 2°C, and the relative humidity was kept at 55%. The mulberries and fruit stalks were taken from the mulberry garden of Southwest University. When we shook the branches vigorously, the mulberries were divided into four categories according to their falling condition and maturity. Including normal mature fruit stalk (MN), mature easy-to-drop fruit stalk (MD), normal young fruit stalk (YN), and easy-to-drop young fruit stalk (YD). At the 2-leaf stage, mulberry seedlings were sprayed with 100 mM ABA and 10 mg/L 2,4-D (auxin hormone regulator). The control group was treated with water. The leaf samples were collected at 0, 1, 3, 6, 12, and 24 h, respectively, and immediately frozen in liquid nitrogen. All samples were subjected to three biological replicates. Mulberry variety “Hongguo No.2” stem was used for lignin staining and paraffin sections.

### 2.2 Identification of *Morus alba* GH9 genes

The *M. alba* genome database (version ASM1206604v3) was downloaded from the NCBI database (https://www.ncbi.nlm.nih.gov/). After this, the GH9 family (PF00759) domain sequence file was retrieved from the Pfam database (Finn et al., 2008) (http://pfam-legacy.xfam.org/) by searching for the keyword “Glyco_hydro_9”. Using this sequence file, we constructed a local database of white mulberry and searched for candidate members of the GH9 gene family using blastp. The presence of complete GH9 domain was checked using hmmer (https://www.ebi.ac.uk/Tools/hmmer/) and NCBI-CDD (https://www.ncbi.nlm.nih.gov/Structure/cdd/wrpsb.cgi). Redundant and incomplete sequences were removed manually. Moreover, isoelectric point (PI) and molecular weight (MW) of *M. alba GH9* proteins were accessed using the ExPASy database (http://www.expasy.org/tools/). The *in silico* analysis of subcellular location, signal peptides, and transmembrane domains was conducted using the Plant-mPLoc server (http://www.csbio.sjtu.edu.cn/bioinf/plant-multi/), SignalP 5.0 (https://services.healthtech.dtu.dk/services/SignalP-5.0/), and TMHMM 2.0 (https://services.healthtech.dtu.dk/services/TMHMM-2.0/), respectively. Sequences of GH9 members of poplar (*Populus* L.) and tomato (*Solanum lycopersicum* L.) were downloaded from the NCBI (https://www.ncbi.nlm.nih.gov/). Sequences of GH9 members of *Arabidopsis* were downloaded from the *Arabidopsis thaliana* database (https://www.arabidopsis.org/index.jsp).

### 2.3 Characterization of GH9 sequences, phylogenetic tree construction, cis-element analysis, chromosome localization and collinearity analysis among species

The internal Muscle program of MEGA version 11.0 software (Tamura et al., 2021) was used to perform multiple sequence alignment of the identified *M. alba* GH9 protein sequences, and a non-root phylogenetic tree with 1,000 bootstrap repeats was constructed using the neighbor-joining (NJ) method. Similarly, another phylogenetic tree was generated among GH9 proteins of *M. alba, A. thaliana, Solanum lycopersicum*, and *Populus tremula* for studying phylogenetic relationships. The 2,000-bp upstream promoter sequence of each gene was analyzed using the PlantCARE (Rombauts et al., 1999) (http://bioinformatics.psb.ugent.be/webtools/plantcare/html/) website for cis-acting regulatory element analysis. Conserved motifs were analyzed using the Motif Elicitation program (http://meme-suite.org/tools/MEME), searching up to 10, and the number of repetitions was arbitrary (Bailey et al., 2009). For the exon–intron structure analysis of the mulberry GH9 coding sequence, their corresponding DNA sequence was used. Gene conserved structure information was obtained from NCBI-CDD (https://www.ncbi.nlm.nih.gov/Structure/cdd/wrpsb.cgi), and then TBtools (Chen et al., 2020) was used to map the gene structure. Gene structure information and chromosome location information (Jiao et al., 2020) were extracted from genome structure annotation files and displayed with TBtools software. Collinearity analysis was performed between mulberry and other species, and the results were plotted using TBtools with MCScanX (Wang et al., 2012).

### 2.4 RNA extraction and quantitative real-time PCR analysis

Total RNA was isolated from all collected samples using Trizol reagent (Invitrogen, USA) according to the manufacturer ‘s protocol. Extracted RNA (1 μg) was reverse transcribed into cDNA using the PrimeScript™ RT kit with gDNA Eraser (TaKaRa, Japan). Primer Premier 5.0 was used to design the specific primers and Primer Blast (https://blast.ncbi.nlm.nih.gov/Blast.cgi) program was used to further verify the specificity of all primers in the NCBI database. qPCR was performed on an ABI Quant studio 3 RealTime system (Applied Biosystems, USA) using SYBR ^®^ Green RealTime PCR Master Mix (Novoprotein, China) with the following reaction program: denaturation (95°C 30 s), amplification and quantification (95°C 3 s, 60°C 30 s, 40 cycles), and melting curve analysis (60–95°C, heating rate 0.3°C/s). Actin gene was used as internal reference. The relative expression levels of genes were calculated by the 2^−ΔΔCT^ method. Statistical analyses were performed using Student’s *t*-test (*t*-test), with three technical replicates established for each qPCR method. Relevant figures were drawn using TBtools and prism 8.0.

### 2.5 Determination of the subcellular localization of MaGH9B6

The cDNA sequence of *M. alba GH9B6*, without a stop codon, was inserted into the 2300-YFP vector using homologous recombination. The resulting construct was then transformed into *Agrobacterium tumefaciens* GV3101. Two sets of *A. tumefaciens* were used to infect the leaves of 1-month-old *N. benthamiana* seedlings: one containing the vector with the fused protein sequences, and the other containing an empty vector (positive control). After 48 h, the infected leaves were observed for fluorescence using a Zen 2 (Olympus) confocal microscope.

### 2.6 Phloroglucinol staining and GUS staining

To prepare the sample, the base of the fruit stalk of the mulberry was cut off near the bark. A 2-cm section of the fruit stalk tissue was manually cut longitudinally. The resulting slice was then positioned on a glass slide, and phloroglucinol staining solution was applied. After staining for 3 min, 25% concentrated hydrochloric acid was added dropwise for 2 min. Subsequently, the sample was examined and photographed under a ZEISS stereomicroscope. For GUS gene expression, the 1,689-bp upstream promoter sequence of *MaGH9B6* was cloned into the PVCT024 vector using homologous recombination. The leaves of 1-month-old *N. benthamiana* seedlings were infected with *Agrobacterium* containing the vector with GUS coding sequence under the control of the MaGH9B6 promoter, and the *Agrobacterium* containing the GUS coding sequence under the 35S promoter (positive control). Wild-type *N. benthamiana* leaves without infection were used as a negative control. Treatments consisted of 24 h exposition to dark, 24 h exposition to light, spraying of solutions containing 100 mM ABA, 10 mg/L 2,4-D, 100 μM gibberellic acid (GA3), 100 mg/L ethylene, and water (control). After 48 h, take 1 cm^2^ of infected leaves with a blade for staining with 40 mM X-gluc (0.2 mL of 0.5 M EDTA, 0.3 mL of 40 mM X-Gluc, 0.1 mL of 10% Triton X-100, and 1 mL of 1 M sodium hydrogen phosphate buffer) dye at 37°C under vacuum for 24 h and then decolored with alcohol. We selected evenly stained leaves for observation and repeated the procedure several times to get the results. The samples were observed and photographed under a stereomicroscope (ZEISS, German).

### 2.7 Paraffin section

Samples of the mulberry stalk (5 mm length) were fixed in 4% paraformaldehyde with 1% glutaraldehyde at 4°C for 24 h. Samples were then dehydrated in an ethanol series and embedded in paraffin prior to cutting 10-μm sections. Sections were stained for morphological observation using 1% (w/v) Safranin O (Amresco, Solon, USA) and 1% (w/v) Fast Green FCF (Merck, Overijse, Belgium) (Zou et al., 2011). Then, they were observed and photographed under a stereomicroscope (ZEISS, German).

## 3 Results

### 3.1 Genome-wide identification of nine members of the GH9 family in *Morus alba*

With the downloaded Glyco_hydro_9 (PF00759) and the reported GH9 as two queries, the *M. alba* protein database was established, and nine non-redundant sequences were finally determined by local blast search (**Supplementary Table 1**). Based on the affinity with other species, GH9 members were named MaGH9A1, MaGH9A2, and MaGH9B1 to MaGH9B7. All GH9 family members were randomly distributed on eight chromosomes and contain 495–1,114 amino acid residues with a molecular weight of 54.52–123.69 kDa. MaGH9B4 had two conserved domains, Chalcone_2 (PF16035.8) and Glyco_hydro_9 (PF00759). Two members (GH9A1 and GH9A2) had transmembrane domains of 75–97 and 77–98 amino acids respectively. However, all members of the GH9B subfamily had signal peptides of 23–34 amino acids except MaGH9B4. This is consistent with the prediction results of previous studies on the GH9 family (Urbanowicz et al., 2007).

### 3.2 The *Morus alba* GH9 gene family has the closest evolutionary relationship with *Populus tremula*

A phylogenetic tree was constructed among *M. alba* GH9 members for evolutionary history analysis. Based on evolutionary relationships, MaGH9 members can be divided into two subgroups: A and B. There are two and seven members in group A and B, respectively.

**Figure 1:**
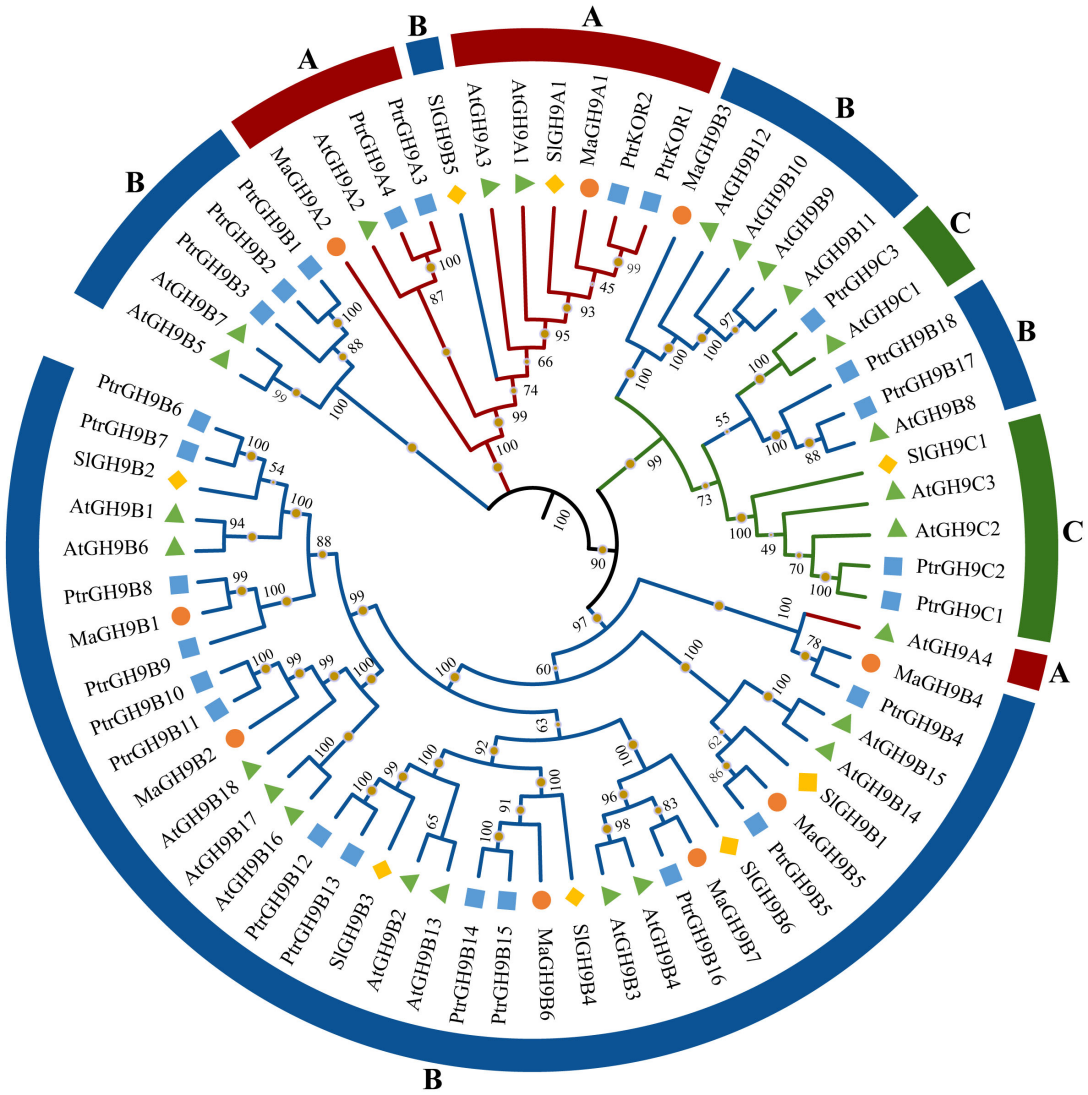
Phylogenetic analysis of 67 GH9 proteins from *Arabidopsis*, tomato, poplar, and mulberry. Diamonds, pyramids, circles, and squares denote tomato, *Arabidopsis*, mulberry, and poplar proteins, respectively. GH9 proteins belonging to subfamilies A, B, and C are highlighted in red, blue, and green, respectively.

According to the phylogenetic tree among *Arabidopsis*, poplar, tomato, and mulberry GH9 members, there were more genes in subgroup B (**Figure 1**). There were 11, 49, and 7 genes in subgroups A, B, and C, respectively. MaGH9 members were more closely related to the members of *P. tremula* GH9 genes. However, the number of GH9 family members in *Populus* was nearly triple that of *M. alba*. This could be attributed to variations in the evolution of GH9 proteins in the two species, as well as differences in the evolution of their chromosomes.

### 3.3 The structure of *Morus alba* GH9 gene family is conservative

**Figure 2:**
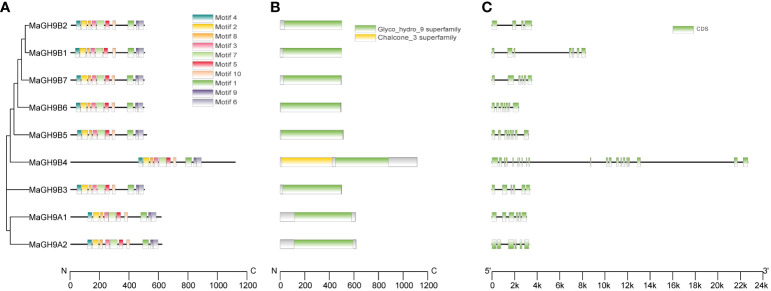
Phylogenetic relationships, conserved motif distribution, glycoside hydrolase domain distribution, and gene structure of *mulberry* GH9 proteins. **(A)** Phylogenetic tree and conserved motif analysis. The boxes with different colors represent 10 different conserved motifs. **(B)** Distribution of glycoside hydrolase domain in the mulberry GH9 protein sequence. The green structure represents glycoside hydrolase domain, the yellow structure represents chalcone_3 domain. **(C)** Exon/intron structure of white mulberry GH9s. The green box and black line indicate exon and intron, respectively. The scale at the bottom denotes the length.

Gene structure analysis showed that the number of exons in the GH9 family ranges from 4 to 21, and most of the glycoside hydrolase structural domains have four to nine exons. Members of subfamily B exhibit a significant variation in gene structure, which may correspond to their complex and diverse functions. The structural domain of glycoside hydrolase is distributed over four to nine exons in subfamily B. On the other hand, subgroup A glycoside hydrolases mainly consist of five exons that distribute the structural domains (**Figures 2B, C**). Motif analysis showed that all the 10 identified conserved motifs were found only in the glycoside hydrolase domain, and these motifs were highly similar in the A and B subfamilies. This indicates that the glycoside hydrolase domain is very consistent in *M. alba* (**Figure 2A**). The N-terminal members of subfamily A have transmembrane domains as they are membrane-bound proteins. On the other hand, six members of subfamily B are predicted to have signal peptides, which suggests that they may be secretory proteins.

### 3.4 *Morus alba* GH9s play a role in hormone signaling pathway

**Figure 3:**
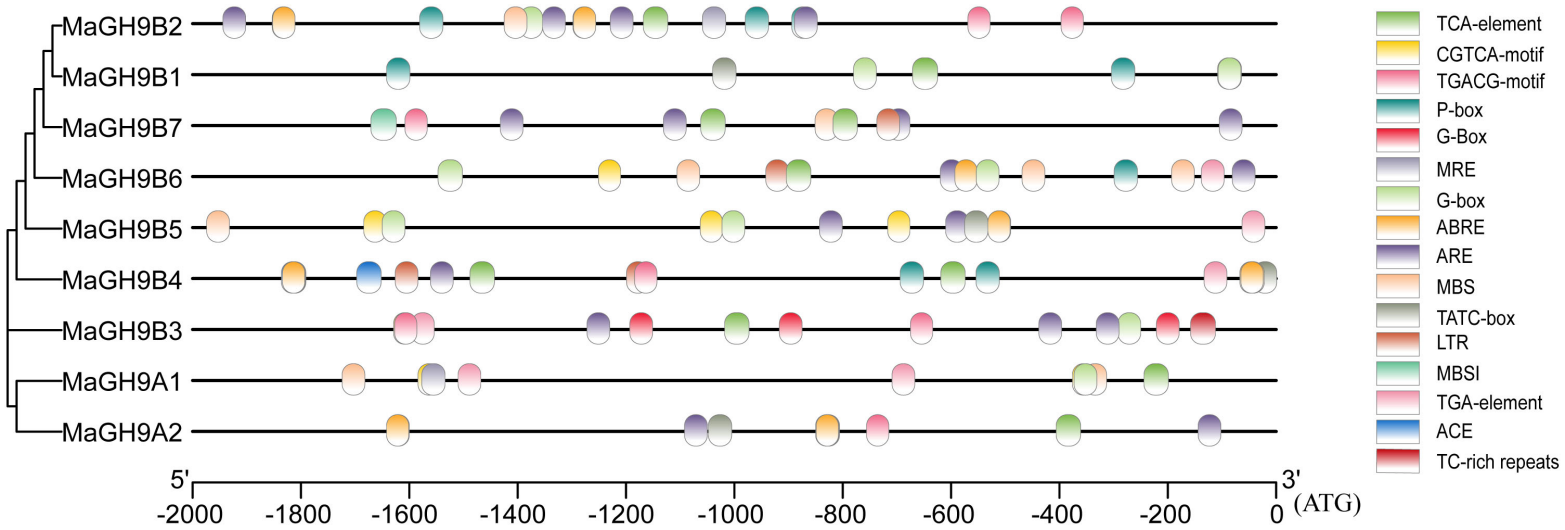
Cis-element prediction in GH9 gene promoters. Different colored boxes denote different cis-elements and the bottom scale denotes position.

Several hormone (salicylic acid, methyl jasmonate, gibberellin, ABA, and auxin) and stress-related cis-elements were predicted (**Supplementary Table 2**). The cis-elements were unevenly distributed in different genes (**Figure 3**). These results suggest that mulberry GH9 members might have critical roles in hormone stress signaling pathways.

### 3.5 The variation of lignin content in mulberry fruit stalks is related to the development of AZ

**Figure 4:**
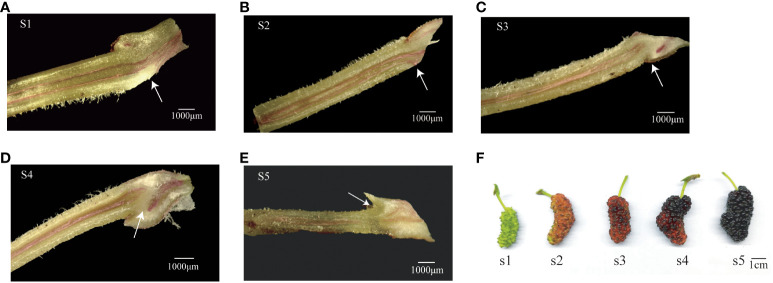
Mulberry fruit stalks stained with phloroglucinol. The arrow points to the abscission zone. **(A)** S1 period, **(B)** S2 period, **(C)** S3 period, **(D)** S4 period, **(E)** S5 period, and **(F)** color change of mulberry in each period.

Cellulase has an adsorption effect on lignin and can effectively degrade lignin content (Lan et al., 2020; Zhao et al., 2022). We stained the fruit stalks with phloroglucinol at five stages of mulberry fruit ripening such as green, green to red, red, red to purple, and purple, which were named S1, S2, S3, S4, and S5, respectively (**Figure 4F**). The lignin part in the fruit stalk was stained red, and the lignin-free part was not stained (**Figures 4A–E**). Lignin in the AZ of the carpopodium appeared as a continuous strip in the S1 and S2 stages. From the S3 stage, the lignin content in the AZ of the carpopodium decreased, forming a phenomenon of lignin deposition far away from the AZs. These findings indicate that the variation in lignin content signifies the developmental stages of the AZ, a process intricately linked to plant flower and fruit drop and closely associated with the GH9 family.

### 3.6 The AZ is formed from the S3 stage

**Figure 5:**
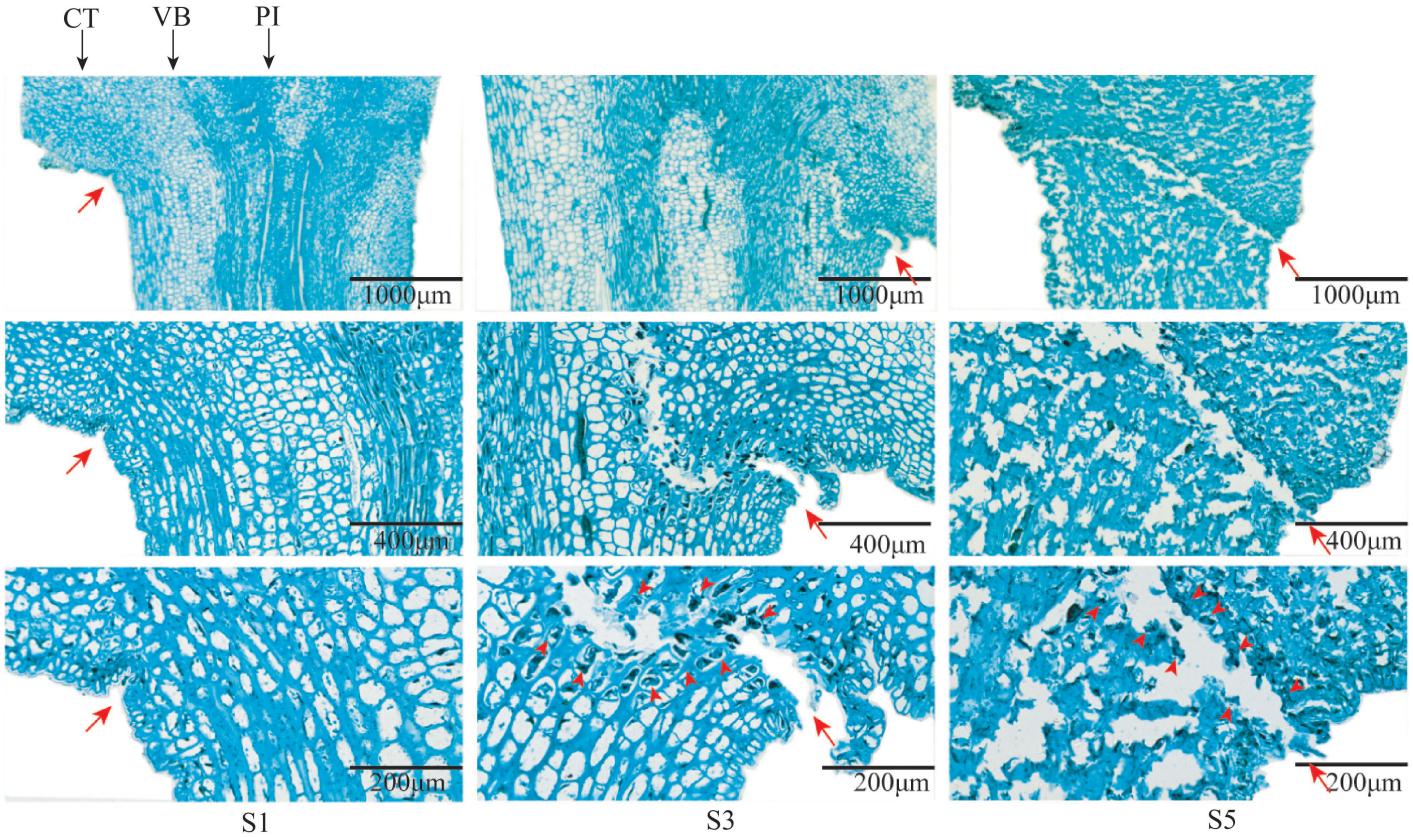
Longitudinal section of AZ of mulberry fruit stalk at S1, S3, and S5 stages. Branches stained with Safranin O and Fast Green. Red arrows indicate the location of the abscission layers. Black arrows indicate the following: CT, cortex; PI, pith; VB, vascular bundle.

The observation of AZ of fruit stalks of mulberry “Hongguo No.2” at S1, S3, and S5 stages by paraffin section showed that there was no AZ in the carpopodium at the S1 stage and a layer of dense cell is present. From S3, the AZ began to appear from the cortical layer and extended to the medulla, and the color of the cells in the AZ began to darken and shrink, forming a scar in the AZ. At S5, the AZ formed a penetrating structure and the cells began to rupture (**Figure 5**).

### 3.7 Subcellular localization and expression analysis of MaGH9B6

**Figure 6:**
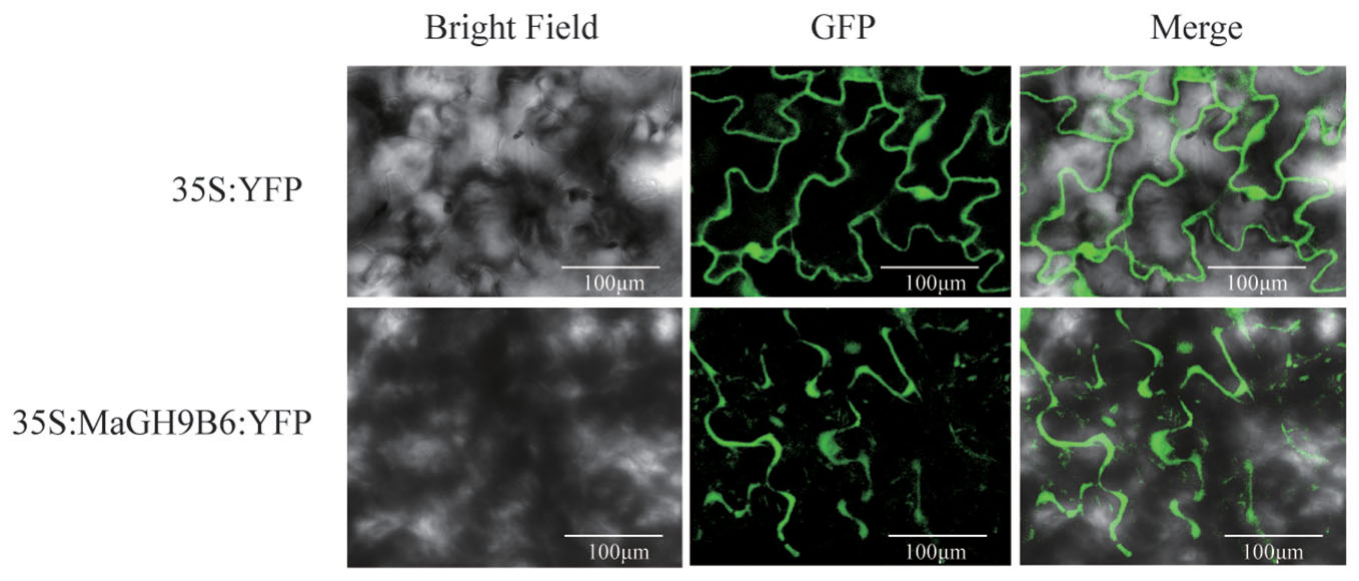
Subcellular localization in *N. benthamiana* analysis of MaGH9B6. The green color represents the GFP signal.

We examined the expression patterns of GH9 genes at various fruit developmental stages, including normal mature fruit stalk (MN), mature easy-to-drop fruit stalk (MD), normal young fruit stalk (YN), and easy-to-drop young fruit stalk (YD). We studied the expression patterns of GH9 gene at these stages. The primers used are listed in **Supplementary Table 3**. The results displayed significant differences in the expression levels of *MaGH9A1*, *MaGH9B1*, *MaGH9B5, MaGH9B6*, *MaGH9B3*, and *MaGH9B7*. Moreover, *MaGH9B1*, *MaGH9B5*, *MaGH9B6*, and *MaGH9B3* exhibited significantly higher expression levels in MD stalks compared to those in MN during the same period. Additionally, the expression level of *MaGH9A1, MaGH9B6*, and *MaGH9B7* in YD was significantly higher compared to that in YN (**Supplementary Figure 1**). Understanding the formation of the AZ is important in exploring the mechanisms behind plant organ abscission. Our investigation into the subcellular localization of MaGH9s, combined with previous research indicating that cellulase genes are predominantly localized to the cell wall and cell membrane, contributes to a comprehensive understanding of these processes (Yan and Wu, 2013; Li et al., 2019b). These results predict the role of this family in the cell wall and related different biological pathways. Based on the results, MaGH9B6 was selected for further study and cloned. It was transiently expressed in the epidermal cells of *N. benthamiana* leaves, and the fluorescence signal was observed. The results showed that the empty vector-controlled GFP signal was seen in both the nucleus and cytoplasm, and MaGH9B6-controlled GFP signals accumulate in the cell wall (**Figure 6**). This finding supports the potential function of MaGH9B6 in cell wall degradation.

### 3.8 Analysis of *MaGH9B6* promoter response to different hormone activity and expression changes following ABA and 2,4-D treatments

**Figure 7:**
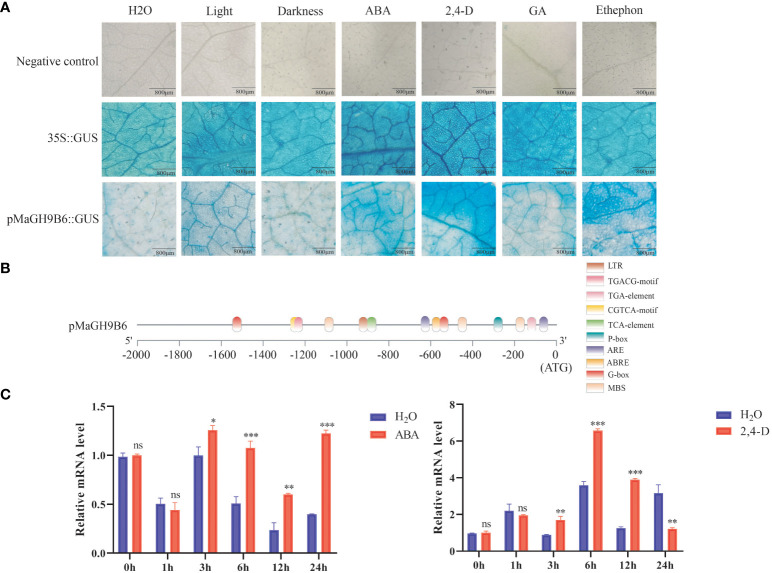
GUS staining of *N. benthamiana* leaves and analysis of *MaGH9B6* under ABA and 2,4-D stress treatments. **(A)** Promoter activity under different hormone treatments. The degree of blue staining of the leaves indirectly reflected the promoter activity. **(B)** *MaGH9B6* promoter structure analysis. **(C)** *MaGH9B6* expression analysis under ABA and 2,4-D stress treatments. Asterisks denote significance level (∗*p* < 0.05, ∗∗*p* < 0.01, ∗∗∗*p* < 0.001, ns means no significant difference).

*In silico* promoter analysis of all genes predicted their involvement in multiple hormone and stress signaling pathways. To get a better understanding, the 1,689-bp upstream sequence of *MaGH9B6* was cloned. X-gluc was used as colorimetric substrate. Transient expression of GUS gene in *N. benthamiana* leaf epidermal cells was assessed through staining after treatment with water, light, darkness, ABA, 2,4-D, ethylene and gibberellin, respectively. Three replications were performed and the *Cauliflower mosaic virus* (CaMV) 35S promoter was used as a positive control and those injected with injection buffer were used as a negative control (**Figure 7A**). As compared to the water-treated group, groups following light-treated, gibberellin, ABA, and 2,4-D treatments were all stained in shades of blue. These results are consistent with *in silico* analysis of the *MaGH9B6* promoter (**Figure 7B**). The *MaGH9B6* promoter showed more response to ABA and 2,4-D, suggesting its response to these hormones.

Furthermore, in 2-month-old mulberry seedlings following 100 mM ABA and 10 mg/L 2,4-D, the quantitative analysis of mulberry leaves showed that *MaGH9B6* expression began to increase significantly from 3 h after ABA stress and continued to increase until 24 h compared to the control. Under 2,4-D stress treatment, *MaGH9B6* expression also increased significantly after 3 h, but the expression level decreased significantly at 24 h compared to the control (**Figure 7C**).

## 4 Discussion

The potential roles of GH9s in other species rationalize the need of genome-wide identification and functional characterization of GH9s in mulberry. Nine GH9s genes were identified and divided into two subgroups based on their phylogenetic relationships: subgroup A and subgroup B. There were more genes in subgroup B. Our results are in line with the previous findings in poplar (Du et al., 2015) and suggest the close evolutionary relationships of these two woody plants. Additionally, the phylogenetic tree and collinearity analysis suggest a close genetic relationship between mulberry and poplar, indicating similar functions and evolutionary history (Jiao et al., 2020; Li et al., 2021). All members of the GH9B subfamily except MaGH9B4 have a C-terminal signal peptide, consistent with previous findings, strengthening the validity of our results (Urbanowicz et al., 2007).

Mulberry is a polyploid perennial plant and is considered to have 14 basic chromosome numbers (Venkatesh et al., 2013; Jiao et al., 2020). Nine members of the GH9 family were randomly distributed on 8 out of 14 chromosomes, suggesting that the involvement of GH9 members in multiple biological processes has different key roles (**Supplementary Figure 2**). All the genes in the MaGH9 family, except *MaGH9B4*, contain the glycoside hydrolase structural domain throughout their coding region, suggesting that the family is functionally conserved. Further gene analysis revealed that two genes in MaGH9s were a result of segmental duplication events, and no tandemly duplicated genes were identified (**Supplementary Figure 3**). These results are supported by previous findings in other crops like poplar (Du et al., 2015) and maize (Buchanan et al., 2012). *MaGH9A1, MaGH9B6*, and *MaGH9B7* showed a significantly higher expression level in the immature fruit stalk, while *MaGH9B1*, *MaGH9B3, MaGH9B5*, and *MaGH9B6* displayed significantly increased expression in the mature fruit stalks, suggesting that these four genes might have key roles in mulberry fruit drop process.

The plant GH9 (glycoside hydrolase 9) family consists of endo-β-1,4 glucanases, which hydrolyze polysaccharides with a β-1,4 glucan backbone, are involved in cellulose biosynthesis, play an important role in cell wall biosynthesis and remodeling, and also play an important role in fruit abscission (He et al., 2018; Li et al., 2019a). Different from bacteria, the potential substrates of plant GH9 genes are cellulose in the amorphous region and non-crystalline polysaccharides (such as xyloglucan). It has been shown that cellulase can adsorb on lignin as a substrate (Tu et al., 2009; Li et al., 2022a) and digest into polysaccharides (Li et al., 2022b). However, the deposition of lignin at the fruit stalk has an important relationship with the fruit drop process and the formation of the AZ (Merelo et al., 2017). We found that the decrease of lignin content in the AZ of the mature fruit stalk of mulberry may be accompanied by the high expression of some *Ma*GH9s members, and we found that one of these, MaGH9B6, was subcellularly localized in the cell wall. This may be related to the potential role it plays in the cell wall.

Promoter analysis of MaGH9 genes found cis-elements having multiple roles in hormonal and stress signaling; i.e., *MaGH9B3*, *MaGH9B4*, *MaGH9B5*, *MaGH9B6*, and *MaGH9A1* contained both ABRE and TGA element (auxin response element). *MaGH9B6* experimental verification corroborated these findings and demonstrated that treatment with ABA and 2,4-D stress significantly increased the gene’s promoter activity. qRT-PCR analysis of *MaGH9B6* in mulberry seedlings at different time points after hormone stress showed that the expression of *MaGH9B6* significantly increased at the early stage in response to 2,4-D and subsequently decreased at the later stage. However, the expression of *MaGH9B6* increased continuously under ABA stress. Endo-1,4-β-glucanase (CEL1) showed higher activities in young and vigorous growing tissues of *Arabidopsis* (Shani et al., 2006). After harvesting, ABA enhanced cellulase activity by regulating cell metabolism to soften blueberry fruits (Zhou et al., 2021), as well as enhanced cellulase activity in cotton leaves following AZ ABA treatment (Mishra et al., 2008). Based on our results and previous findings, our speculation suggests that *MaGH9B6* might play a role in responding to auxin during the early stages of mulberry growth and development, aiding in the formation of the tree’s epidermis and xylem. Later on, its function might involve responding to the regulation of ABA, which is related to cell division, flower development, and fruit drop.

In summary, we performed detailed genome identification and bioinformatic analysis of the GH9 family in mulberry. Moreover, some MaGH9 genes displayed different transcript accumulation in stalks at different fruit developmental stages and with a different tendency to drop fruits. The change of lignin content in the fruit stalk could indirectly reflect the change of cellulase activity in different periods, and could also be implied in the process of fruit abscission. *MaGH9B6* showed differential expression at different time points against different hormonal treatments, i.e., ABA and 2,4-D. These results suggest the response of MaGH9 genes against hormonal treatments and new candidate genes for mulberry breeding. However, further studies are needed to have an in-depth understanding of the regulation mechanism of fruit drop in mulberry.

## Data availability statement

The original contributions presented in the study are included in the article/**Supplementary Material**. Further inquiries can be directed to the corresponding authors.

## Author contributions

JD: Conceptualization, Data curation, Formal analysis, Methodology, Software, Writing – original draft, Writing – review & editing. BA: Writing – review & editing. XD: Methodology, Writing – review & editing. ZF: Methodology, Writing – review & editing. LL: Methodology, Writing – review & editing. XL: Methodology, Writing – review & editing. YP: Writing – review & editing. XZ: Conceptualization, Data curation, Funding acquisition, Methodology, Validation, Writing – review & editing.
