# Supplementary material for: Genome-wide analysis of the mulberry (Morus abla L.) GH9 gene family and the functional characterization of MaGH9B6 during the development of the abscission zone
Source: Front Plant Sci. 2024 Apr 3;15:1352635. doi: 10.3389/fpls.2024.1352635 (PMC11021789; doi:10.3389/fpls.2024.1352635)
Supplement: Supplementary file 1 [file DataSheet_1.pdf]

Supplementary Material

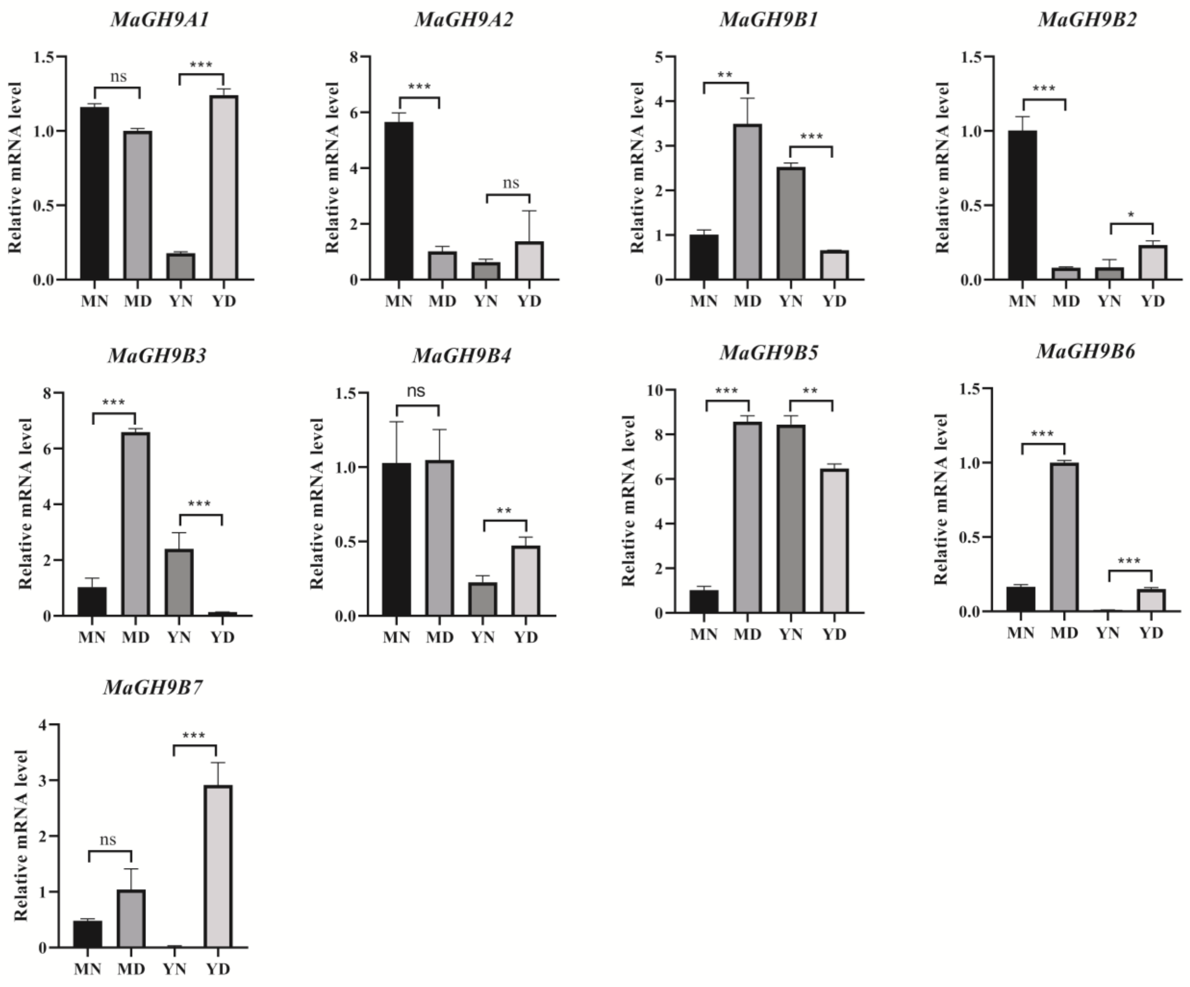

Supplementary Figure 1. Histogram of expression profile of *Morus alba* GH9s at different fruit developmental stages and with different tendency to drop fruits. MN， normal mature fruit stalk; MD, mature easy-to-drop fruit stalk; YN, normal young fruit stalk, YD, easy-to-drop young fruit stalk. Asterisks denote significance level (∗$p < 0.05$, ∗∗$p < 0.01$, ∗∗∗$p < 0.001$, ns means no significant difference)

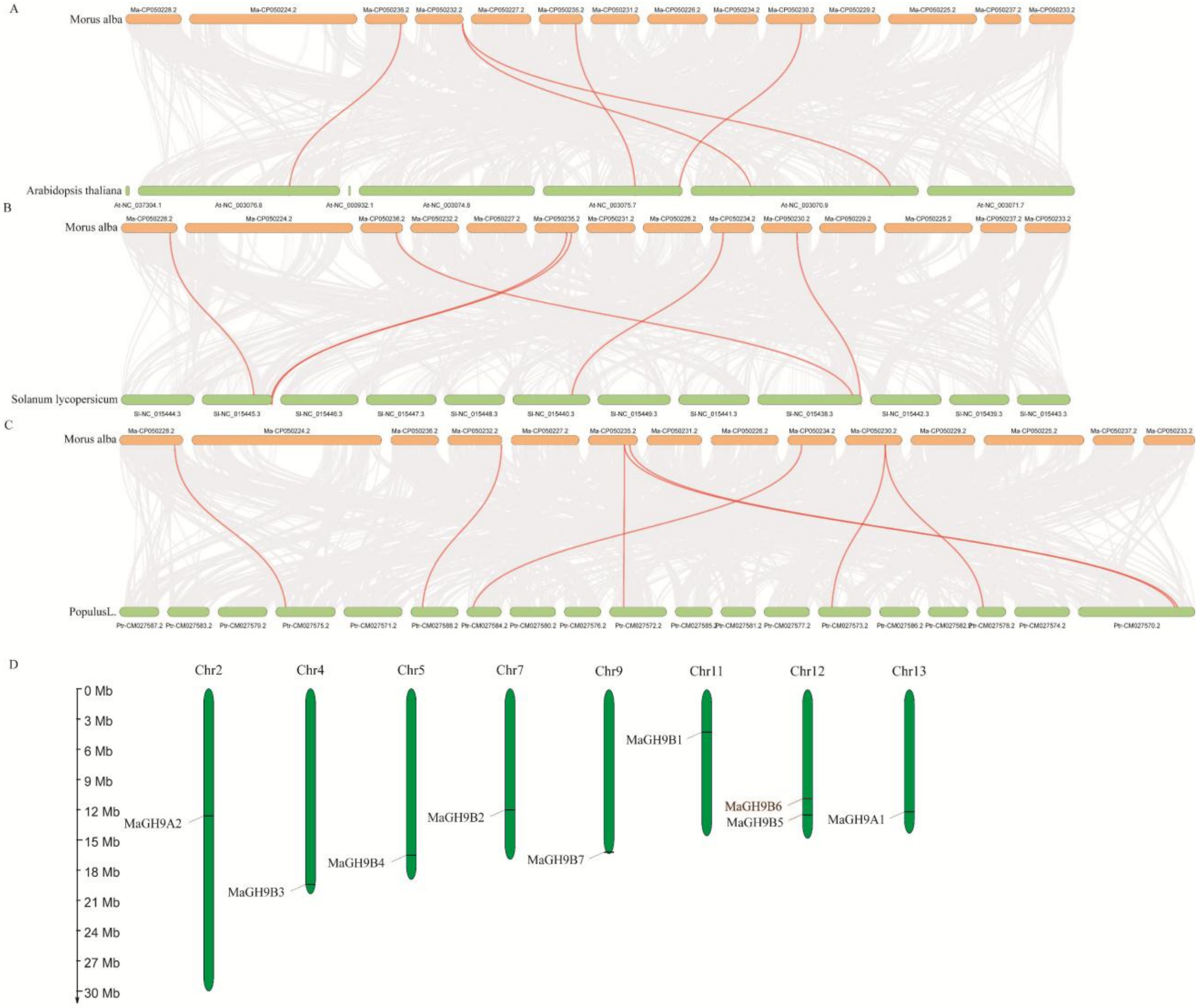

Supplementary Figure 2. Collinearity analysis and chromosome location of GH9 proteins. (A) Mulberry is covalently related to the *Arabidopsis* GH9 gene family, (B) Mulberry is covalently related to the tomato GH9 gene family, and (C) Mulberry is covalently related to the poplar GH9 gene family. Red highlighted lines indicate genes with colinearity. (D) Chromosomal positions of mulberry GH9 genes.

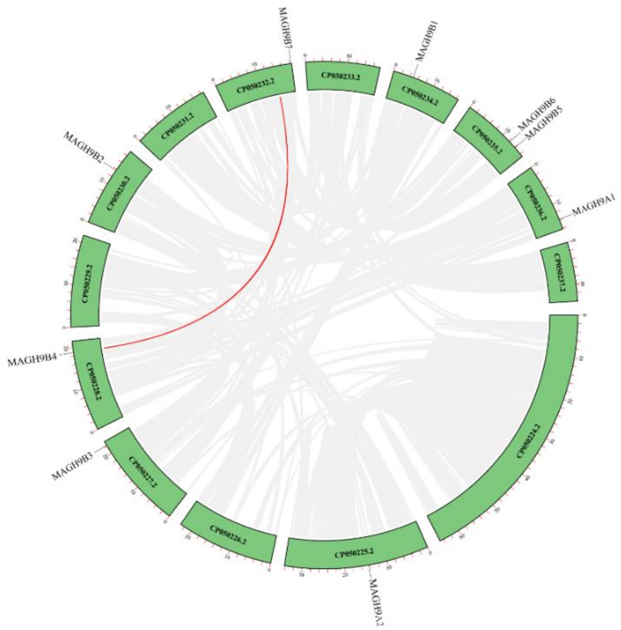

Supplementary Figure 3. Intraspecific collinearity analysis.

Gene name	Accession ID	Protein length	Protein molecular weight MW/kD	Isoelectric point PI	Subcellular localization	Signal peptide/aa	Corresponding accession number of Morus notabilis
MaGH9A1	MALBA024705.1	612	68.23	8.98	Cell membrane.	——	XP_010088712.1
MaGH9A2	MALBA004476.1	617	68.52	6.26	Cell membrane.	——	XP_024028547.1
MaGH9B1	MALBA020963.1	500	55.05	7.12	Cell membrane. Cell wall.	1～25	XP_024018060.1
MaGH9B2	MALBA015007.1	501	55.62	4.79	Cell membrane. Cell wall.	1～27	XP_024030152.1
MaGH9B3	MALBA009123.1	499	55.53	6.28	Cell membrane. Cell wall.	1～34	XP_024019832.1
MaGH9B4	MALBA011106.1	1114	123.69	8.60	Cell membrane.	——	XP_010098378.1

MaGH9B5	MALBA023225.1	513	56.18	6.02	Cell membrane. Cell wall	1～23	XP_024031981.1
MaGH9B6	MALBA023038.1	495	54.52	6.23	Cell membrane. Cell wall.	1～27	XP_010100207.1
MaGH9B7	MALBA018686.1	497	55.16	9.30	Cell wall.	1～24	XP_010098378.1

Supplementary Table 1. Physical and chemical properties and subcellular localization of GH9 family proteins in *Morus alba*.

Component name	Core sequence (5 '-3')	Characteristic
TCA-element	CCATCTTTTT/TCAGAAGAGG	Cis-acting elements involved in the salicylic acid reaction
CGTCA-motif	CGTCA	Cis-regulatory elements involved in MeJA response
TGACG-motif	TGACG	Cis-regulatory elements involved in MeJA response
P-box	CCTTTTG	Gibberellin response element
G-Box/G-box	TACGTG/CCACGTAA/CACGTC/TAACACGTAG	Cis-regulatory elements involved in photoresponse
MRE	AACCTAA	MYB binding sites involved in 、photoreaction
ABRE	ACGTG/GACACGTGGC/CACGTG	Cis-acting element involved in abscisic acid reaction
ARE	AAACCA	Cis-regulatory elements essential for anaerobic induction

MBS	CAACTG	MYB binding sites involved in drought induction
TATC-box	TATCCCA	Cis-acting elements involved in gibberellin response
LTR	CCGAAA	Cis-acting element involved in low-temperature reaction
MBSI	TTTTTACGGTTA	MYB binding sites involved in Gene Regulation of Flavonoid Biosynthesis
TGA-element	AACGAC	Auxin response element
ACE	GACACGTATG/CTAACGTATT	Cis-acting element involved in light reaction
TC-rich repeats	ATTCTCTAAC	Cis-acting elements involved in defense and stress response

Supplementary Table 2. *Morus alba* GH9 gene family promoter cis-acting element.

Gene ID	Forward primer	Reverse primer
qMaGH9A1	ATTCTACCGATGTACTGCGTGAT	ATGGAAACCGTCGTGCTTAT
qMaGH9A2	GCACCAAACATAGAAAGACG	GGGAGCGAATCATAAGCAGT
qMaGH9B1	ATTTCAACGGCTACCAGGAT	GAAGGAAGCAGAAGTAGAGGG
qMaGH9B2	GATTCCCGATGGCATTCACC	TTGAGCAGGTAGTCGGTGGC
qMaGH9B3	CTGCCTCGCAACCAAAGAGT	AAATGCCATCGGAAACCCTA
qMaGH9B4	TGTCGTCAAACTCGGTGGTG	GTTGTCGTAGCGGTCAGGTC
qMaGH9B5	CTGGCTATCAGGATGAGTTGT	GGCTCCACCCTTTGTTGCTT
qMaGH9B6	AATCTCACAGGCAAAGAAACA	GTATGGACGGCACAGAGGA
qMaGH9B7	GTCCGTTTATTCGGTCTCAG	CACAGCAGCTCATCCTTGTAT

Supplementary Table 3. List of gene-specific primers used in the present study.
